# Swine Flu: Knowledge, Attitude, and Practices Survey of Medical and Dental Students of Karachi

**DOI:** 10.7759/cureus.2048

**Published:** 2018-01-09

**Authors:** Fariha Hasan, Mohammad O Khan, Mukarram Ali

**Affiliations:** 1 Department of Internal Medicine, Dow University of Health Sciences (DUHS), Karachi, Pakistan; 2 Department of Forensic Medicine and Toxicology, Dow University of Health Sciences (DUHS), Karachi, Pakistan

**Keywords:** swine flu, knowledge attitude practice, influenza a, medical students, influenza h1n1, dental students, swine influenza

## Abstract

Introduction

Pakistan is extremely susceptible to an influenza outbreak, as it shares borders with the most affected countries, namely China and India. The medical and dental students come into direct contact with the affected population and should be aware of the risk factors and signs and symptoms pertaining to swine influenza virus (SIV). Hence, this survey was conducted to assess the knowledge, perceptions and self-care practices of the medical and dental students with regards to this pandemic.

Methods

A descriptive, cross-sectional study was conducted to evaluate the swine flu-related knowledge, attitudes and practices of the medical and dental students at various institutions in Karachi, Pakistan. We approached 613 students that were available on the dates of this survey, keeping a medical to dental student ratio of 75:25. All students from first to final year comprised of the study population, and no internists or medical personnel were included. The questionnaire was divided into three sections, namely knowledge, attitudes and, practices. All questions were based on a multiple choice format. The data were entered and interpreted using the IBM Statistical Package for the Social Sciences 23.0 (IBM Corp., Armonk, New York).

Results

The majority of the students were aware that the swine flu is a transmittable disease (n=485, 80.8%). Most students identified the signs and symptoms correctly; however, diarrhea (15.5%) and vomiting (32.2%) were the least correct answers (n=93, n=193 respectively). Most of the preventative measures were reported accurately by the participants. Despite this, only 15.5% students (n=93) reported the use of a facemask when suffering from fever, cough and a runny nose.

Conclusion

There is a dire need for the routine integration of the awareness and management programs in the medical and dental schools. There exists a gap between the policy and practice, and it is high time we bridge the divide. The students should also be vaccinated annually for influenza A.

## Introduction

Claiming the lives of approximately 50-100 million people, the Spanish flu pandemic caused by the H1N1 subtype of influenza A virus alarmed the world by its immense pandemic potential [1-2]. One of the factors that make these organisms a considerable threat is the frequent occurrence of minor point mutations in their genetic material, resulting in different strains of viruses [3]. After multiple outbreaks throughout the 20th century, perhaps one of the most striking events occurred in 2009 when a novel H1N1 virus emerged; this resulted in the swine flu pandemic, that by June 2009 had caused the World Health Organization (WHO) to raise its pandemic alert level to phase six [4]. It was later estimated by the Centers for Disease Control and Prevention (CDC), that the global death toll from this pandemic was more than 284,000, which was around 15 times higher than the laboratory confirmed cases [5-6].

One of the countries that are susceptible to influenza outbreaks is Pakistan. Multiple outbreaks in different parts of the country have been reported, notably in the provinces of Punjab and Sindh, with substantial deaths occurring due to the disease [7-9]. Even with robust vaccination efforts being made, it was not surprising to find two doctors contracting the disease [10], while Lahore (a major city in Pakistan) was recently added to the list of cities reporting the swine flu cases [11]. Given Pakistan’s condition as a developing country, its lack of health care facilities and ignorance regarding public health matters, it is not unreasonable to suspect that the actual number of the cases must have been higher than those reported.

There are several factors making Pakistan extremely susceptible to a future pandemic. Perhaps the most obvious one corresponds to Pakistan sharing significant borders with China and India, both of which, are countries with very large populations, high pig densities and a large number of reported cases. Furthermore, the provinces of Pakistan most affected by the disease, namely Punjab and Sindh, share borders with the three of the most affected Indian provinces of Rajasthan, Gujarat, and Punjab [12]. Additionally, thousands of Pakistanis visit the Kingdom of Saudi Arabia for holy pilgrimages throughout the year and can easily be infected by swine influenza virus (SIV) from Muslim carriers of different origins. Additionally, the China-Pakistan Economic Corridor (CPEC) is a project that promises to bring in an influx of the Chinese and Central Asian investors, tourists, laborers, and health workers into Pakistan, which will increase the transmission of this disease in major metropolises of the country [13].

A developing country like Pakistan lacks the basic technical and diagnostic facilities to report and treat SIV cases, and in the occurrence of such an event, Pakistan might not be able to tackle it as well as other countries. The medical and dental students, the diagnosticians of tomorrow, come into direct contact with the affected population and should be aware of the risk factors and the signs and symptoms pertaining to SIV. Hence, we conducted a survey taking into account the medical and dental students, as both groups are directly exposed to the infected individuals during the clinical practices. The dental students are particularly susceptible, due to their close proximity to open mouths and hence transmission via respiratory droplets. Previous studies have been conducted on dental students [14], but so far, no study encompasses both the medical and dental students. The compliance with preventive measures can only increase as a result of increased awareness. A previous survey performed by Khowaja, et al. [15] evaluated the baseline knowledge of the medical students of Karachi in 2009. Since then, measures have been taken by both the government and private organizations to address this public health issue [16]. Hence, this survey was conducted to assess the knowledge, perceptions and self-care practices of the medical and dental students. We also aimed to reassess the works of previous surveys, and consequently gauge if our findings were in line with those of the former studies. The secondary objective of the study was to determine the factors contributing to a low perceived risk. We believe that by establishing the baseline knowledge and practices regarding SIV, we can generate preliminary data that can subsequently be used to focus and guide public health interventions.

## Materials and methods

A descriptive, cross-sectional study was conducted to evaluate the swine flu-related knowledge, attitudes and practices of the medical and dental students at various institutions in Karachi, Pakistan. A sample size of 387 was calculated using OpenEpi.com; however, 613 students were included in this study to get a better representation of the population. We approached 613 students that were available on the dates of this survey. In total, 13 participants failed to complete the survey, giving a response rate of 97.9%. The study was executed after the approval was received by the Institutional Review Board of the Dow University of Health Sciences.

The medical and dental students of the government and private colleges, including the Dow University of Health Sciences and the Jinnah Postgraduate Medical Centre, were selected using non-probability convenience sampling, spanning a time period of one month from August 2017 to October 2017. A validated and pre-tested questionnaire was formed [17], and distributed amongst 20 students as a convenience based pilot test. The ambiguous questions and questions regarding the mythical concepts were omitted from the final performa. To minimize bias and improve understanding, two doctors reviewed the questionnaire. All students from the first to the final year comprised the study population, and no internists or medical personnel were included. All those who hesitated to participate were excluded.

The sociodemographic details of each student included the age, gender, year of study and course of study. The questionnaire was divided into three sections, namely knowledge, attitudes, and practices. All questions were based on a multiple choice format. The first part focused on the nature of the disease, mode of transmission, signs, and symptoms, risk factors, incubation period, availability of the medication, vaccines and possible complications. The second part was based on practices and was assessed by the students' hand hygiene and use of a face mask. The attitudes were gauged by the perception of the participants regarding a swine flu outbreak and contact with an infected patient.

The data were entered and interpreted using the IBM Statistical Package for the Social Sciences 23.0 (IBM Corp., Armonk, New York). A knowledge score was calculated to reflect the participants' overall knowledge regarding the risk factors and signs/symptoms. This was scored out of a total of 31 points. The frequencies and percentages were calculated for the categorical responses. The Chi-squared tests with 95% confidence interval were applied to see whether there was any statistical difference between the knowledge of the medical and dental students, in order to assess which group needs to be better prepared for a potential health crisis. The differences in continuous variables, such as knowledge score, with respect to the categorical variables such as 'gender', 'course of study' and 'year of study' were assessed using non-parametric tests. A p-value of less than 0.05 was deemed significant.

## Results

In total, 600 out of the 611 participants who were distributed the questionnaire returned it with complete data that was included in the analyzes, yielding a response rate of 98.2%. The mean age of the respondents was 20 ± 1.41 years. The basic demographic details of the study participants are presented in Table 1.

**Table 1:** The demographic characteristics of the respondents.

Measure	No. of participants n=600 (%)
Area of study	
Medical	450 (75.0)
Dental	150 (25.0)
Age (years)	
17-20	399 (66.5)
21-26	201 (33.5)
Gender	
Male	156 (26.0)
Female	444 (74.0)

Table 2 shows the knowledge score of the participants and co-relates it to the 'gender' and 'course of study'. Additionally, there was no significant difference in the knowledge found between 'age', 'gender', 'year of study' or 'course of study'.

**Table 2:** The overall knowledge scores of the participants and scores stratified by the demographic characteristics.

Demographic characteristics	Knowledge score	p value
Overall	15.97 +/- 6.00	
Males	15.97 +/- 6.66	p > 0.05
Females	15.97 +/- 5.80	
Medical	16.10 +/- 5.92	p > 0.05
Dental	15.57 +/- 6.24	

Table 3 assesses the knowledge of the participants.

**Table 3:** The knowledge about swine flu disease. Correct answers to this question were counted under the column yes and incorrect under no. The p-value signifies the difference in knowledge between medical and dental students.

Statement	Yes (%)	No (%)	Don’t know (%)	p-value
1. Is swine flu a respiratory disease?	362(60.3)	70(11.7)	168(28.0)	p > 0.05
2. Can viruses cause swine flu?	525(87.5)	14(2.3)	61(10.2)	p > 0.05
3. Is swine flu a transmittable disease?	485(80.8)	31(5.2)	84(14)	p > 0.05
4. Which of the following are symptoms of swine flu?				
a) Fever and chills	434(72.3)	19(3.2)	147(24.5)	p = 0.015
b) Cold, cough and sore throat	432(72.0)	27(4.5)	141(23.5)	p > 0.05
c) Diarrhea	93(15.5)	166(27.7)	341(56.8)	p > 0.05
d) Muscle fatigue	294(49.0)	58(9.7)	248(41.3)	p = 0.048
e) Vomiting	193(32.2)	83(13.8)	324(54.0)	p > 0.05
f) Headache	292(48.7)	42(7.0)	266(44.3)	p > 0.05
5. Can diagnosis of swine flu be confirmed by laboratory testing of a respiratory sample?	307(51.2)	51(8.5)	242(40.3)	p> 0.05
6. Which of the following are the potential modes of transmission of swine flu?				
a) Air borne like coughing, sneezing	452(75.3)	22(3.7)	126(21.0)	p=0.000
b) Touching an infected person	168(28.0)	162(27.0)	270(45.0)	p > 0.05
c) Using objects of an infected person	282(47.0)	70(11.7)	248(41.3)	p=0.03
7. Can swine flu virus be transmitted from:				
a) Pigs to humans	380(63.3)	45(7.5)	175(29.2)	p=0.002
b) Humans to humans	362(60.3)	37(6.2)	201(33.5)	p > 0.05
8. Have you been vaccinated for swine flu?	10(1.7)	377(62.8)	213(35.5)	p=0.001
9. Is there any medication available for swine flu?	276(46.0)	90(15.0)	234(39.0)	p=0.000
10. Can swine flu cause death?	100 (10.0)	390(65.0)	110(18.3)	p >0.05
11. Is swine flu recurrent in human beings?	183(30.5)	108(18.0)	309(51.5)	p=0.036
12. Are humans supposed to wear gloves while working with sick animals to prevent transmission?	423(70.5)	47(7.8)	130(21.7)	p > 0.05
13. What are the possible complications of swine flu?				
a) Respiratory failure and death	410(68.3)	30(5.0)	160(26.7)	p=0.006
b) Bacterial or viral pneumonia	317(52.8)	49(8.2)	234(39.0)	p > 0.05
c) Exacerbation of previous illness	202(33.7)	69(11.5)	329(54.8)	p > 0.05
d) Neurological symptoms	118(19.7)	130(21.7)	352(58.7)	p > 0.05
14. What are the preventive methods of human to human transmission?				
a) Washing hands	429(71.5)	29(4.8)	142(23.7)	p > 0.05
b) Wearing a mask	467(77.8)	27(4.5)	106(17.7)	p > 0.05
c) Avoid touching eyes, nose, and mouth	342(57.0)	63(10.5)	195(32.5)	p > 0.05
d) Restricting cough and sneeze with tissue	450(75.0)	21(3.5)	129(21.5)	p=0.039
15. After being infected, how long does it take for the symptoms to appear?*	55(9.2)	545(90.8)	-----------	p=0.027

Of this cohort, 2.3% of the students had previously encountered a swine flu patient and 1.8% claimed to have suffered the disease. Regarding the causative organism of the disease, a minority of the students falsely believed that it is caused by a bacterium (7.8%) or it is an inherited condition (4.7%). The medical students were more likely to correctly identify the symptoms, including fever and chills (p=0.015) and muscle fatigue (p=0.048) compared to the dental students, while vomiting was a symptom better identified by people who had suffered from swine flu disease (p=0.024), relative to those who had not. A few students thought that disease transmission could occur through the sexual intercourse (10.2%) or mosquito bites (14.3%), and more than one-quarter of them believed it could occur through contaminated food and water (39.0%) like eating cooked pork (35.2%). The people who had previously encountered a swine flu patient identified the contact with an infected person (p <0.001) and using the belongings of an infected person (p=0.017) as potential modes of transmission better than those who had not encountered a swine flu patient. The people who had suffered from swine flu were more likely to identify placing a tissue on their mouth and nose when sneezing and coughing as a measure to prevent human to human transmission (p=0.022). Compared to the dental students, the medical students had greater knowledge of the transmission mode, that is from pigs to humans (p=0.002), via the air (p <0.001) and using an infected persons' belongings (p=0.03). The medical students were more likely to identify the availability of the medications and vaccinations for swine flu (p <0.001 and p=0.001, respectively).

The preventative measures practiced by the participants were assessed and are presented in Table 4.

**Table 4:** The self-care practices.

Statement	Yes (%)The self-care practices.	No (%)	Sometimes (%)
1. When coughing or sneezing:			
a) Do you cover your mouth and nose with a tissue or handkerchief?	466(77.7)	40(6.7)	94(15.7)
b) Do you throw away the used tissue into the bin?	511(85.2)	26(4.3)	63(10.5)
c) Do you turn your face from the surrounding people?	510(85.0)	28(4.7)	62(10.3)
2. Do you wash your hands:			
a) Before touching your eye and nose?	193(32.2)	227(37.8)	180(30.0)
b) After covering the nose while sneezing?	402(67.0)	75(12.5)	123(20.5)
c) After using the toilet?	549(91.5)	17(2.8)	34(5.7)
3. Do you apply soap while washing your hands?	509(84.8)	17(2.8)	74(12.3)
4. Regarding your usage of facemask:			
a) Do you wear a facemask when having fever, cough or a runny nose?	93(15.5)	425(70.8)	82(13.7)
b) Do you wear a facemask in crowded places?	69(11.5)	462(77.0)	69(11.5)
c) Do you change the facemask after using it once?	233(38.8)	269(44.8)	98(16.3)
d) Do you never use a face mask?	306(51.0)	212(35.3)	82(13.7)
5. Regarding a person infected with swine flu:			
a) You avoid contact with an infected person	483(80.5)	44(7.3)	73(12.2)
b) You avoid touching and shaking hands	431(71.8)	83(13.8)	86(14.3)
c) Put handkerchief on nose and mouth when around them?	407(67.8)	110(18.3)	83(13.8)

The questions related to the preventive measures showed that the medical students, compared to the dental students, were more likely to wash their hands before touching their eyes and nose (p=0.004), to wear a facemask in crowded areas (p <0.001) and to change the facemask after it has been used once (p <0.001). The students who had suffered from swine flu and had a previous encounter with a swine flu patient were more likely to practice putting a handkerchief over their nose and mouth when around an infected person (p=0.002 and p=0.002, respectively). The participants who believed that swine flu was a recurrent disease were more likely to visit a doctor upon experiencing symptoms of the disease (p=0.013).

Table 5 illustrates the attitude responses of participants.

**Table 5:** The attitude towards a swine flu outbreak.

Statement	Yes	No	p-value
After getting to know about a swine flu outbreak:			
a) Do you wash your hands more frequently than before?	408 (68.0)	192 (32.0)	p > 0.05
b) Do you seek for additional information regarding swine flu?	413 (68.8)	187 (31.2)	p > 0.05
c) Consume a more nutritional diet than before?	290 (48.3)	310 (51.7)	p = 0.011
d) Drink plenty of water?	346 (57.7)	254 (42.3)	p = 0.006
e) Do you avoid going to crowded places?	315 (52.5)	285 (47.5)	p = 0.009

The people who thought of swine flu as a transmittable disease were more likely to seek the additional information upon learning about a swine flu outbreak (p=0.012), avoid contact with an infected person (p <0.001) such as touching and shaking hands with them (p=0.015), apply soap while washing their hands (p=0.019) and wash their hands after using the toilet (p=0.041). The people who perceived swine flu as a fatal disease were more likely to wear a facemask in crowded areas (p=0.036) and wash their hands after using the toilet (p=0.023).

## Discussion

Our study has several key findings. Three quarters (75.3%) of our students were able to correctly identify coughing, sneezing and talking as the major modes of transmission of the virus. This finding mirror was that of Hussain, et al. [18] and he disagrees with the findings of Khowaja, et al. [15]. Although knowledge of the participants regarding the signs and symptoms and modes of transmission seemed decent, this did not translate into practice. The majority of the students agreed that the use of a facemask is an effective preventative measure, but only a minute percentage of the students agreed to use a facemask when sick. This statistic is disappointing and contrasts with the results of Hussain, et al. [18], who showed the appropriate use of a facemask by students. These findings also reflect upon the gap between the policy and the implementation of the guidelines.

Regarding the symptoms of infection, most of the common flu-like symptoms such as fever, cough, and fatigue were correctly identified by a majority of the participants. However, vomiting and diarrhea, which are specific to SIV, were seldom mentioned correctly. This finding is in line with that of Hussain, et al. [18] and indicates the inability to correctly recognize and diagnose a swine flu patient by our participants. This, in turn, leads to the lack of protective measures and facilitates transmission of the virus.

Our study shows that the risk perception of the participants was low. This result is in line with previous surveys [19-20]. Most respondents agreed that the disease is transmittable; however, they did not believe that they could contract the virus. This is reflected by the negligible number of students vaccinated against the virus (1.7%); this reported low number coincides with a previous survey [21]. The findings of May, et al. [20] are contradictory to ours as they described high vaccination rates amongst the medical students and residents. The lack of importance given to the swine flu vaccines is obvious and paints the institutions in a bad light. All the medical and dental schools should ensure prior vaccination before the start of every academic year and maintain this practice throughout. The aforementioned low perceived risk can also be assessed by the nonchalant attitude of the students towards 'wanting to seek additional information about the swine flu'. However, this perception can be explained by the low number of the confirmed cases in Pakistan [22] and the breeding of pigs being uncommon in the country; hence, the major mode of transmission is eliminated. It is concerning to note that only 67.8% of the respondents agreed to wear a facemask when around the infected individuals. The medical and dental students are in constant contact with affected patients as part of their clinical curriculum, and can inadvertently transmit the virus to immunocompromised individuals. This unethical practice should be stopped and hospitals should make sure that the affected medical and dental students be given a leave of absence.

Hand washing and the compliance with sanitary practices such as consuming a nutritional diet in case of a swine flu outbreak seemed inadequate. This contradicts the findings of Lau, et al. [23] and suggests negligence by the government. The campaigns regarding the preparedness against the outbreaks through the use of leaflets, posters and public health broadcasts on television and social media [23] are some of the ways to promote personal hygiene and consequently increase the compliance with preventative measures. This is of particular concern as hand washing and the use of a facemask by the medical and dental students has the potential to become a norm in our society and subsequently serves an important determinant of better health outcomes.

Despite our best efforts, there are limitations to our study. Firstly, the sample size is small and did not represent the medical and dental students in the entire country. Secondly, we used convenience sampling to collect responses and so our study suffered from the selection bias. Thirdly, the responses from only two schools were included in our study due to the lack of resources. Consequently, the extrapolation of the data to represent the entire country is difficult. Furthermore, the students also had hectic routines and this may have affected their responses negatively, especially if they did not deem the survey to be mandatory and were not getting any sort of compensation for their time.

## Conclusions

Although the morbidity and mortality of this infection are very low, an outbreak of SIV in Pakistan is inevitable due to our population living in close proximity to the affected countries. There is a need for routine integration of the awareness and management programs in the medical and dental schools. The students should regularly be vaccinated against influenza. Furthermore, we urge the healthcare professionals to practice preventative measures pertinent to controlling future outbreaks. We hope that this study can pave the way for surveys to be conducted on a larger scale, encompassing the medical and dental students of the entire country.
